# Clinical Value of Machine Learning in the Automated Detection of Focal Cortical Dysplasia Using Quantitative Multimodal Surface-Based Features

**DOI:** 10.3389/fnins.2018.01008

**Published:** 2019-01-11

**Authors:** Jia-Jie Mo, Jian-Guo Zhang, Wen-Ling Li, Chao Chen, Na-Jing Zhou, Wen-Han Hu, Chao Zhang, Yao Wang, Xiu Wang, Chang Liu, Bao-Tian Zhao, Jun-Jian Zhou, Kai Zhang

**Affiliations:** ^1^Department of Functional Neurosurgery, Beijing Tiantan Hospital, Capital Medical University, Beijing, China; ^2^Department of Functional Neurosurgery, The Second Hospital of Hebei Medical University, Shijiazhuang, China; ^3^Key Laboratory of Complex System Control Theory and Application, Tianjin University of Technology, Tianjin, China; ^4^Department of Pharmacology, Hebei Medical University, Shijiazhuang, China

**Keywords:** focal cortical dysplasia, machine learning, metabolic, morphological, quantitative

## Abstract

**Objective:** To automatically detect focal cortical dysplasia (FCD) lesion by combining quantitative multimodal surface-based features with machine learning and to assess its clinical value.

**Methods:** Neuroimaging data and clinical information for 74 participants (40 with histologically proven FCD type II) was retrospectively included. The morphology, intensity and function-based features characterizing FCD lesions were calculated vertex-wise on each cortical surface and fed to an artificial neural network. The classifier performance was quantitatively and qualitatively assessed by performing statistical analysis and conventional visual analysis.

**Results:** The accuracy, sensitivity, specificity of the neural network classifier based on multimodal surface-based features were 70.5%, 70.0%, and 69.9%, respectively, which outperformed the unimodal classifier. There was no significant difference in the detection rate of FCD subtypes (*Pearson’s Chi-Square* = 0.001, *p* = 0.970). *Cohen’s kappa score* between automated detection outcomes and post-surgical resection region was 0.385 (considered as fair).

**Conclusion:** Automated machine learning with multimodal surface features can provide objective and intelligent detection of FCD lesion in pre-surgical evaluation and can assist the surgical strategy. Furthermore, the optimal parameters, appropriate surface features and efficient algorithm are worth exploring.

## Introduction

Focal cortical dysplasia (FCD) was intrinsically epileptogenic and was a significant cause of medically refractory epilepsy (Fauser, 2015). FCD had been reported as being increasingly frequent in a series of patients who had undergone epilepsy surgery and was the most common histopathological diagnosis among children (Blumcke et al., 2017). For patients in whom FCD lesions were focal, epilepsy surgery may be an option. Complete resection of the FCD lesions, including surrounding epileptogenic areas, correlated with a satisfied prognosis and fewer complications (Timoney and Rutka, 2017). Therefore, accurate detection of the localization and extent of epileptogenic lesions during pre-surgical evaluation was crucial because it affected not only surgical decisions, but also the intracranial electroencephalogram (iEEG) implantation strategy when the lesions were located in highly functional areas (e.g., speech, motor skills) (Chassoux et al., 2017).

Focal cortical dysplasia constituted a broad spectrum of histopathological and clinical features ranging from FCD type I (small or subtle in conventional magnetic resonance imaging [MRI]) to FCD type III (severe pathology with other associated epileptogenic lesions) (Blümcke et al., 2011). Radiologically, the features of FCD included the following: (1) local cortical thinning or thickening; (2) blurring of the gray-white matter (GM/WM) boundary; (3) gyration anomalies; (4) abnormal signal intensity on fluid-attenuated inversion recovery (FLAIR)/T2-weighted MRI (including the *Transmantle* sign in FCD IIb); (5) abnormal interhemispheric asymmetry in structural patterns; (6) lobar hypoplasia/atrophy; and (7) diffuse or multifocal occurrence in any of the above features (Lee et al., 1998; Bernasconi, 2003; Colombo et al., 2012). Additionally, ^18^fluoro-2-deoxy-d-glucose (^18^FDG) positron emission tomography–computed tomography (PET-CT) was performed to help with the localization of epileptogenic disturbances in metabolism, which may aid the identification of occult FCD that were missed on MRI. PET-CT often revealed focal hypometabolism in the FCD region and has been shown to have a diagnostic sensitivity of 78–83% in FCD detection (Chassoux et al., 2010; Yh et al., 2011). The accuracy increased further with the use of PET/MRI co-registration (Salamon et al., 2008). Despite enormous progress in neuroimaging techniques and computational methods, many lesions remain subtle to identify, as the sensitivity is approximately 70% of patients with FCD (Wang et al., 2013; Kini et al., 2016). Approximately 30% of patients with visually negative MRI cause inherent difficulty in identifying the epileptogenic zone (EZ). Furthermore, in some cases, re-examination of MRI images indicates that lesions were missed during initial interpretation, and the pre-operative evaluation process was time-consuming and depends upon the experience of the interpreters, which may hinder the localization of the EZ and advancements of surgical treatments.

To overcome the limitations of radiological assessment of FCD, quantitative computational analysis and machine learning methods have built a series of feature measures into an identification algorithm to improve the detection rate (Adler et al., 2017; Hong et al., 2017; Jin et al., 2018; Tan et al., 2018). For example, morphometric analysis on T1-weighted MRI was designed to generate *z*-score maps to identify the abnormal extension of the GM/WM boundary and GM/WM junction (Wong-Kisiel et al., 2018); voxel-based 3-dimensional (3-D) MRI analysis evaluated FCD by voxel-wise subtraction of the mean GM density map of the normal database, and the resulting dataset is searched for local and global maxima (Kassubek et al., 2010); an automated algorithm was trained on MRI-negative patients with histologically confirmed FCD to improve the diagnostic accuracy (Hong et al., 2014); and quantification of the ^18^F-FDG PET could help identify subtle lesions as a complement to the visual analysis (Mendes et al., 2017). To date, developing an accurate diagnostic tool that combines machine learning and quantitative imaging features, to identify potential epileptogenic foci was expected.

Our overall approach was to combine machine learning methods with quantitative multimodal surface-based features for the automated detection of FCD lesions. First, we included eligible structural and functional images [T1-magnetization prepared rapid gradient echo (T1-MPRAGE) sequence, T2-FLAIR sequence and PET], performed image pre-processing [space standardization, cortical construction, co-registration and drawing the region of interest (ROI)] and extracted surface features (morphological and metabolic characteristics). Then, we configured a machine learning model by choosing an appropriate algorithm and defining related parameters, training the model with labeled feature inputs and making predictions on the new dataset. Finally, we evaluated the clinical value of this method both quantitatively and qualitatively by performing statistical analysis and conventional visual analysis.

## Materials and Methods

**FIGURE 1:**
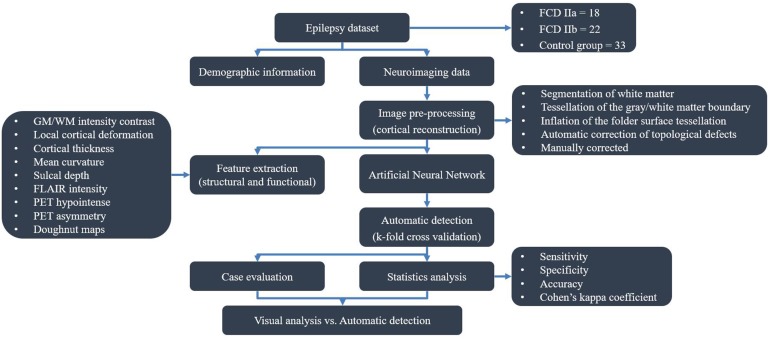
Flow diagram of study design.

The flow diagram outlining the study design and results is shown in Figure 1.

### Participants

Forty participants with medically refractory epilepsy who had undergone pre-surgical assessment in the Epilepsy Centre of Beijing Tiantan Hospital between 2015 and 2018 were retrospectively included in the present study. The pathologic diagnosis was confirmed and subtyped according to the International League Against Epilepsy 2010 classification (Kwan et al., 2010). To accurately label the lesion, only the patients with histopathological proven FCD type II and positive imaging were included. To assess specificity and perform inter-subject normalization, we additionally included 33 patients with short duration (less than 3 years) and histopathological confirmation of hippocampal sclerosis (HS) or epidermoid cyst (EC) as control group because it was difficult to find healthy subjects with PET images who were free of central nervous system disease in the clinical setting. In addition, other clinical centers also adapted patients with HS and EC as reference group (Adler et al., 2017; Tan et al., 2018). All patients in control group underwent epilepsy surgery and had histopathological verification of the absence of FCD. Simultaneously, an iEEG was applied to confirm the EZ was located at mesio-temporal regions, in some cases.

All the included participants fulfilled the following inclusion criteria: (1) complete clinical data (including demographic information, origin T1-MPRAGE, T2-FLAIR and PET imaging, and histological diagnosis); and (2) performance of lesionectomy. The following patients were excluded: (1) patients who were less than 3 years old, as the myelination of the neonatal brain does not reach maturity (Soun et al., 2016), which may influence imaging data normalization; (2) patients with low-quality images resulting from head motion, noise or other image artifacts; and (3) patients with FCD III on histopathology, as other principle lesions may affect the performance of the artificial neural network (ANN).

Brain imaging data were visually analyzed by expert neuroradiologists according to established MRI and PET characteristic features (Lee et al., 1998; Bernasconi, 2003; Chassoux et al., 2010; Colombo et al., 2012). The determination of location and border of suspected lesions was validated by an epilepsy multidisciplinary team that consisted of neurosurgeons, neurologists, neurophysiologists and neuropsychologists. The surgery strategy was decided based on pre-surgical evaluation (semiology, structural and functional imaging and EEG data) during an epilepsy surgery meeting. We postulated that the EZ was located inside the resection region, based on seizure improvement following surgery. Surgical outcome data were obtained via direct clinical assessment or telephone interview and were determined based on the International League Against Epilepsy (ILAE) classification system (Wieser et al., 2010).

**FIGURE 2:**
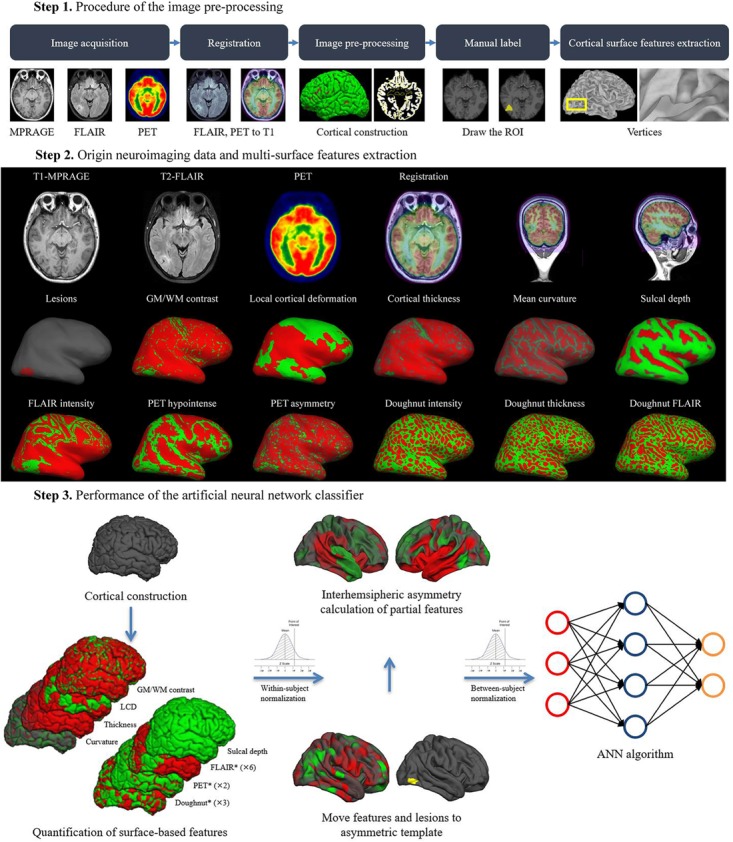
Overall procedure of automatic detection of FCD.

The overall procedure of the automated detection approach is shown in Figure 2.

### MRI and PET Imaging Data

In our centre, all neuroimages were acquired at 3.0 T field strength using dedicated MRI epilepsy protocols, including 3-dimensional (3D) T1-MPRAGE sequence [repetition time (TR) = 2,300 ms, echo time (TE) = 2.53 ms, flip angle = 12°, slice thickness = 1 mm, no gap, voxel size = 1 mm × 1 mm × 1 mm], and axial T2-FLAIR (TR = 7,000 ms, TE = 80 ms, flip angle = 12°, slice thickness = 1 mm, no gap, voxel size = 1 mm × 1 mm × 1 mm). T1-MPRAGE and T2-FLAIR sequences offered advantages in identifying subtle differences in cortical tissue (Viviani et al., 2017). FDG-PET scans were acquired in the interictal state under standard resting conditions (eyes closed, dimmed ambient light). Approximately 45 min following the intravenous administration of ^18^F-labeled FDG, PET images of the brain were obtained from the vertex to the skull base (voxel size was 1 mm, slice thickness was 3.27 mm). Images were attenuation-corrected using non-contrast CT transmission information.

### Pre-processing and Normalization

Pre-processing involved T1-MPRAGE and T2-FLAIR images undergoing automated intensity non-uniformity correction and intensity normalization, which could upgrade the accuracy of registration performance (Bagci et al., 2010). Then, T1-MPRAGE images were linearly registered to the *Montreal Neurological Institute (MNI) 152* symmetric template (Evans et al., 1993). Following that step, T2-FLAIR and PET images were linearly mapped to the T1-MPARAGE images in *MNI* space, and subsequently, analysis was performed using the *SPM12* software package (available for free download^1^ ) (Figure 2).

### Surface Reconstruction

*FreeSurfer* software v5.3^2^ (NeuroImage. Cortical surface-based analysis, 1999; Fischl and Dale, 2000) was used to perform the cortical reconstruction and to register the T2-FLAIR sequence and PET scans to the T1-MPRAGE with the *recon-all* pipeline (NeuroImage. Cortical surface-based analysis, 1999). The pipeline provided a full processing stream for structural images, including the following: (1) motion correction and averaging volumetric T1 weighted images; (2) removal of non-brain tissue; (3) automated *Talairach* transformation; (4) segmentation of the subcortical white matter and deep gray matter volumetric structures; (5) tessellation of the gray matter white matter boundary; (6) automated topology correction; and (7) surface deformation to optimally place the GM/WM and GM/cerebrospinal fluid (CSF) borders at the location. Once the cortical models were complete, a number of deformable procedures could be performed for further data processing and analysis, including surface inflation (NeuroImage. Cortical surface-based analysis, 1999). Additionally, cortical surface data and brain volumes could be displayed in *tksurfer* and *freeview*, respectively. These steps were illustrated in detail in the tutorials and a prior study (Reuter et al., 2012). The results of reconstruction and surface extraction were validated by visual inspection, and any inaccuracies were manually corrected.

### Manual Lesion Masks

Manual lesion labels of FCD were created for 40 patients in *freeview* on an axial T1-MPRAGE volumetric scan. The location of the lesions were confirmed by the outcomes of pre-surgical evaluation (the neuroimaging features in combination with seizure semiology, clinical examination and video EEG). After that, the labels were converted to surface for compatibility with the surface reconstructions and normalized to zero-mean, *MNI* standard space. Non-lesional tissues were sampled from the contralateral, healthy, homotopic cortex.

### FCD Feature Extraction From MRI and PET

Labels of vertex morphologic [GM/WM intensity contrast, local cortical deformation (LGD), cortical thickness, mean curvature, sulcal depth, doughnut intensity, doughnut thickness], intensity (FLAIR intensity at a different level of the cortical depth, doughnut FLAIR) and metabolic (PET hypointense, PET asymmetry) features were calculated. The MRI and PET features were computed after first warping surfaces back into each subject’s MRI native space and subsequently into PET native space by inversely transforming the previously performed PET-to-MRI registration. These features were then resampled on the *MNI* surface template using the related transformation. The technical details of these features were described in a prior study (Adler et al., 2017), and all codes are freely available at https://github.com/kwagstyl/FCDdetection. The measurements of features were represented below.

### Measures of Morphological Features

GM/WM intensity contrast was measured as the ratio of the GM signal intensity to the WM signal intensity (Fauser, 2015; Adler et al., 2017). The GM and WM signal intensities were measured 30% through the thickness of the cortical ribbon and 1 mm below the GM/WM interface, respectively (Salat et al., 2009). FCD lesions with blurring of the GM/WM boundary were expected to have low GM/WM intensity contrast values compared to those of the non-FCD cortex; (Blumcke et al., 2017) LGD was measured as the degree of cortical intrinsic curvature of a 25 mm radius ring that was centered on a vertex (Ronan et al., 2011; Adler et al., 2017; Timoney and Rutka, 2017). The cortical thickness was measured as the shortest distance between corresponding vertices on the GM/WM surfaces (Fischl and Dale, 2000; Chassoux et al., 2017). The mean curvature was measured as the area-minimizing flow that defines the deviation from the cortical surface to a sphere at the GM/WM boundary (Blümcke et al., 2011; Jin et al., 2018) The sulcal depth was measured as the geodesic distance between the given vertices within sulci and the gyral crown vertices (Boucher et al., 2009). As reported in prior studies, small FCD lesions were located at the bottom of a deep sulcus (Hofman et al., 2011).

### Measures of Intensity Features

FLAIR intensities at the GM/WM boundary as well as at 25%, 50%, and 75% of cortical thickness, and at 0.5 mm and 1 mm below the boundary, were sampled. Decreased vertical gradient indicated the blurring of the GM/WM boundary (Hong et al., 2017).

### Measures of Metabolic Features

Details about extraction of PET features can be found elsewhere (Tan et al., 2018). (1) PET hypointensity was measured as normalized intensity at each vertex of the FCD lesion; (2) PET asymmetry was calculated to compare the relatively lower PET intensity in the FCD lesion to that of the homotopic location in the contralateral brain hemisphere.

### Doughnut Map

Comparison of the GM/WM intensity contrast, cortical thickness and FLAIR intensities between a 6 mm radius circle on the inflated surface and the surrounding region around the circle was conducive to identifying the local change and reducing the spurious motion effect (Adler et al., 2017).

### Features Smoothing and Normalization

Prior to classification, all the features were smoothed with a 10 mm full-width-at-half-maximum (FWHM) *Gaussian* surface kernel (Merkx et al., 2012). Then, for each type of feature across all the vertices within a given individual, we would perform the within-subject *z*-score normalization, followed by the between-subject *z*-score normalization, so that feature values at a given vertex were normalized to the control group.

### Interhemispheric Asymmetry

The morphological, intensity, metabolic features and doughnut maps performed the interhemispheric registration on the average space (*fsaverage_sym*). Interhemispheric registration of feature maps allowed quantification of the interhemispheric asymmetry of surface-based metrics at each vertex. An initial template was created from only the left hemisphere value, and bilateral hemisphere values were aligned with this initial left template. A new template was then created from these bilateral surfaces, and the surfaces were reregistered to it. This new template was a mixture of left and right hemisphere and thus was less biased (Greve et al., 2013).

### Performance of Machine Learning

Automated detection of FCD lesion was performed using an ANN classifier implemented in MATLAB R2017b (MathWorks, Natick, MA, United States). The neural network classifier was trained on the aforementioned neuroimaging features sampled from the vertices of the labeled lesion and selected non-lesional vertices. Each vertex in the training data was given two values: “1” for the lesion in the mask; “0” for the non-lesional hemisphere. The feedforward network was widely used and provides a proven method of building a non-parametric classifier. It contained input, hidden and output layers. The non-linear behavior of the hidden and output layers generated classifier behavior (Haykin, 1994). Principal component analysis (PCA) was applied to reduce the input dimension and speed up the learning algorithm. Every node in the proceeding layer took a weighted average of the outputs of the previous layer, until an output was reached. The value of output layers from the weighted sum of inputs determines the property of each vertex. And 70% of the available data was allocated for training. The remaining 30% of data were equally partitioned as validation and test datasets. Feature selection, training, and performance evaluation were carried out using *k*-fold cross validations (*k* = 5) with 100 iterations. At each iteration, the dataset was randomly partitioned into k equal sized subsets. Then, a single subset was retained as the validation data for testing the model, and the remaining k – 1 subsets were used as training data (Rodriguez et al., 2010). The threshold of probability maps at the highest detection and lowest false-positive rates were set in all the classification schemes. The evaluation of classification accuracy was assessed with regards to post-surgical resection regions and the standard-of-care clinical evaluation.

### Statistical Analysis

All numeric data had a non-parametric statistical distribution according to the *Shapiro–Wilk* test. For descriptive data compared between the patient group and control group, *Pearson’s Chi-square test* or *Fisher’s exact test* and *Student t-test* or *Mann–Whitney test* were used. Statistical significance was set at the 5% level. All results were considered as concordant if there was a major positive cluster located at the surgically resected areas. The findings of each diagnostic output were separated into true positive (TP), true negative (TN), false positive (FP), and false negative (FN). A comparison of automated detection outcomes to surgical resection regions were visually determined. TP (also called the detection rate) was defined as the proportion of patients in whom a detected cluster correctly overlapped with the post-surgical resection region. TN was calculated as the proportion of controls in whom no FCD lesion cluster was falsely identified. The sensitivity was calculated as TP/(TP + FN), specificity as TN/(TN + FP) and accuracy as (TP + TN)/(TP + FP + FN + TN). Agreement in correctly identifying the resection area was determined between automated detection outcomes using *Cohen’s kappa scores*. According to a prior study, *kappa* values were classified as slight (0.00–0.20), fair (0.21–0.40), moderate (0.41–0.60), substantial (0.61–0.80), and almost perfect (0.81–1.00) (Landis and Koch, 1977). Statistical analysis was performed with SPSS software, version 20.0.0 (IBM corp., United States).

## Results

### Patient Demographics and Clinical Information

Demographics information and lesion characteristics were summarized in Table 1. In total, seventy-three cases (36 female, 37 male) were eligibly included in the present study. In the patient group, 18 patients (24.3%) had histologically confirmed FCD IIa, 22 (29.7%) had FCD IIb; and in the control group, 32 (43.2%) had HS and 1 (1.4%) had EC. The sex proportion of the control group was not significantly different from that of the patient group (*Pearson’s Chi-Square* = 0.117, *p* = 0.733), nor was the hemisphere lateralization (*Pearson’s Chi-Square* = 0.030, *p* = 0.862). At the same time, there was no significant difference in duration between the each group (*Mann–Whitney U*: *p* = 0.942, as the variables of the patient group did not correspond to a normal distribution). The epilepsy duration ranged from 0.1 to 33 years [mean 11.2 years, standard deviation (SD) 8.3 years] in the patient group and from 0.5 to 32 years (mean 11.3 years, SD 8.3 years) in the control group. Seizure freedom was achieved in 82.5% (33/40) of participants 1 year after surgery.

**Table 1:** Overview of the clinical features of the 73 patients with MRI lesion and pathologic diagnosis.

	Patient group	Control group	*P*-value
Participants	40	33	–
Sex (%)	Female: 19 (47.5%) Male: 21 (52.5%)	Female: 17 (51.5%) Male: 16 (48.5%)	0.733^a^
Duration (mean ± SD, years)	11.2 ± 8.3, range 0.1–33	11.3 ± 8.3, range 0.5–32	0.942^b^
Pathology (%)	FCD IIa 18 (45.0%) FCD IIb 22 (55.0%)	HS 32 (97.0%) EC 1 (3.0%)	–
Hemisphere (%)	Left 21 (52.5%) Right 19 (47.5%)	Left 18 (54.5%) Right 15 (45.5%)	0.862^a^
Lesion location (%)	Frontal lobe 26 (65.0%) Temporal lobe 4 (10.0%) Parietal lobe 5 (12.5%) Occipital lobe 2 (5.0%) Insular lobe 3 (7.5%)	–	–


*FCD, focal cortical dysplasia; HS, hippocampal sclerosis; EC, epidermoid cyst; SD, standard deviation. ^*a*^*Pearson’s Chi-Square* test; ^*b*^*Mann–Whitney U* test.*

### Performance of the Neural Network Classifier (Quantitative Analysis)

**FIGURE 3:**
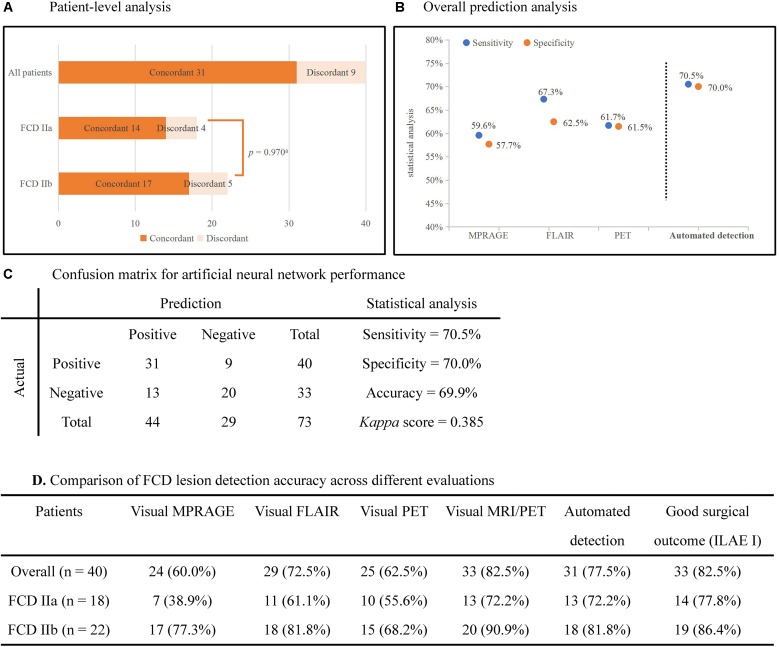
Automated detection outcomes. **(A)** Patient-level analysis. Numbers in histograms represented number of patients. The percentage of patients in whom automated outcomes were concordant with the surgical resection is 77.5% (31/40) in all patients, 72.2% (13/18) in FCD IIa subgroup, and 81.8% (18/22) in FCD IIb subgroup. There was no significant difference between subgroups (*Pearson’s Chi-Square* = 0.001, *p* = 0.970). **(B)** The plot showed the sensitivity and specificity of separate neural networks operating on unimodal and multimodal features. **(C)** Confusion matrix for neural networks showed the outcomes of statistical analysis. **(D)** The detection rates of different images and the automated detection outcomes**. ILAE I:** completely seizure-free without auras in ILAE classification. ^a^*Pearson’s Chi-Square* test.

The statistical analysis of automated detection outcomes is available in Figure 3. According to the final output, the detected clusters colocalized with the post-surgical resection region in 31 patients, yielding a TP of 77.5% (31/40), and 13 lesional clusters were identified in the control group, resulting in 60.6% (20/33) TN. Therefore, according to the aforementioned formulas, the sensitivity was calculated as 70.5%, specificity as 70.0% and accuracy as 69.9%. There was no significant difference in the detection rate of FCD subtypes (*Pearson’s Chi-Square* = 0.001, *p* = 0.970). The outcomes of separate neural networks operating on unimodal (lesional features derived from only one modality, such as T1-MPRAGE, T2-FLAIR or PET) were lower than the performance on multimodal classifiers, and the statistical analysis is shown in Figures 3B,D. *Cohen’s kappa score* between the automated detection outcomes and post-surgical resection region was 0.385 (considered as fair).

### Case Evaluation (Qualitative Analysis)

**FIGURE 4:**
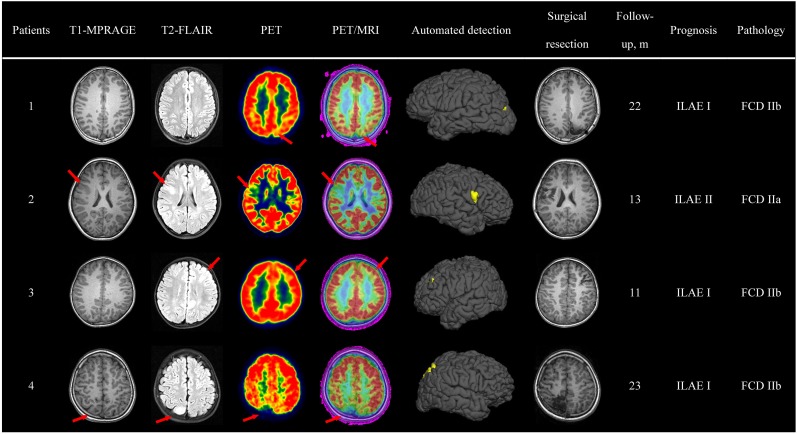
Case evaluation. Examples of automated detection outcomes in four patients with the diagnosis of FCD. Red arrows pointed to the lesion. The absence of red arrows indicated negative diagnosis in the initial report. Evaluation of surgical outcome based on the International League Against Epilepsy (ILAE) classification system.

Pre-surgical imaging data, automated detection outcomes, surgical resection and a follow-up survey are available in Figure 4. Here, we take patient 3 as an example to display the qualitative analysis.

Patient 3, a 4-year-old, right-handed male pediatric patient, started having seizures 3 years ago. His familial and personal history was irrelevant. Seizure symptomatology was characterized by impaired awareness, facial flushing, and upper limb flexion. Seizures occurred more than 10 times per day and lasted about half a minute. Neurologic examination was normal, and neuropsychological evaluation revealed no memory dysfunctions. During video-EEG monitoring, interictal spikes were recorded over the left frontal lobe and central area (C3, F3), and ictal activity appeared in the same areas. The initial MRI report revealed abnormal signs in the left frontal lobe. A careful visual analysis of FDG-PET and registered PET-MRI revealed hypometabolism of the left occipital lobe (bottom of middle frontal gyrus). The outcomes of semiology, EEG and neuroimaging data pointed to the same suspected epileptogenic area; thus, SEEG was unnecessary. A lesionectomy (from the anterior boundary of lesion to the precentral sulcus) was performed, and the patient has remained seizure-free for 11 months, with no neurological deficit. Pathologic examination showed FCD IIb. In addition, the automated detection outcome was concordant with the surgical resection region.

## Discussion

### Achievement

Focal cortical dysplasia caused medically refractory epilepsy and was amenable to surgical treatment (Blümcke et al., 2011). Completeness of resection of the dysplastic tissue and additional epileptogenic tissue was considered to be one critical factor in determining outcome after surgery (Blount, 2017). Even with the most advanced imaging techniques, the subtle radiographic appearance of FCD still rendered visual identification challenging. Meanwhile, the interpretation of neuroimaging was time-consuming, subjective and based on the interpreters’ experience, which may result in erroneous or miss-diagnosis. Clearly, to accurately localize the lesions, achieve better prognosis and minimize the resection of uninvolved regions, an objective and machine-aided diagnostic tool was necessary. Therefore, the aim of the present study was to combine machine learning methods with quantitative neuroimaging features for automated identification of the site and extent of the FCD type II lesion. The neural network classifier performance was evaluated quantitatively and qualitatively by performing statistical analysis and conventional visual analysis. Overall, in the present study, the neuroimaging data and demographic information of seventy-three participants were included to train the neural network classifier. The accuracy, sensitivity, and specificity of the classifier were 70.5%, 70.0%, and 69.9%, respectively. *Cohen’s kappa score* between the automated detection outcomes and post-surgical resection region was 0.385 (considered as fair). There was no significant difference in the detection rate of FCD subtypes. In summary, the proposed method had great potential to become an auxiliary tool for diagnosis of epilepsy in pre-surgical evaluation. We considered that future strategies for exploring optimal parameters, appropriate surface features and an efficient algorithm were worthwhile.

### Correlated Literatures

Currently, an improvement in magnetic field strength (De Ciantis et al., 2016) and sequences of MRI had improved the detection of FCD. Texture analysis (Antel et al., 2003; Besson et al., 2008; Wong-Kisiel et al., 2018) and voxel-based analysis (Huppertz et al., 2001; Kassubek et al., 2010) had been used to identify FCD characteristics in a quantitative fashion. But these methods were criticized for their subjective inspection. In addition, voxel-based methods neglect anatomical relationships across the folded cortex and amplify unwanted partial volume effects, which also led to less remarkable outcomes (Hong et al., 2014). To overcome these drawbacks, Tan et al. (2018) introduced an algorithm reliant on surface-based features that statistically combine morphology, intensity and metabolism and has better performance (Hong et al., 2014). At the same time, as the increasing number and complexity of medical images threatens to overwhelm radiologists’ capacity to interpret them, machine learning provided an effective way to automate the analysis and diagnosis of medical images (Wang and Summers, 2012). Machine learning methods were increasingly popular in imaging diagnosis prognostic estimation. The approaches were able to process enormous amounts of clinical data and perform quantitative analysis to make the conclusion more objective. However, many scientific and practical challenges still needed to be addressed: variation in imaging protocols, weak labels, interpretation of results and so on (Bruijne, 2016). In the present study, we took advantage of the availability of PET scans and expected to achieve superior sensitivity in FCD detection using feature modeling of combined MRI and PET, compared to that using quantitative MRI alone (Hong et al., 2014; Adler et al., 2017; Mendes et al., 2017; Jin et al., 2018). However, our neural network classifier underperformed in identifying the FCD lesion [the detection rate was lower than that in previous work (Adler et al., 2017; Jin et al., 2018; Tan et al., 2018)]. Several reasons may explain this phenomenon. First, multimodal surface features (morphology, intensity, and metabolism) of FCD were extracted to train the classifier. However, only several features were confirmed as valuable, such as cortical thickness and GM/WM matter intensity (Hong et al., 2014; Jin et al., 2018). Therefore, the unsatisfied performance may result from other irrelevant and noisy features (the so-called “overfitting problem”) when we fed all the features to ANN (Hawkins, 2004). Though the cross validation had been used for evaluation, the problem could not be avoided completely. Second, different protocols of available neuroimaging data may have an influence on data consistency. On the other hand, relatively low TN rates (60.6%, 20/33) in the control group suggested that the classifier failed to ignore healthy tissue and disregard FCD-unrelated pathology in the control group. This outcome may also be attributable to the redundant multimodal surface features. Moreover, extra-primary clusters were found in several patients. However, no post-surgical pathology was available, as these regions were not resected. Prior studies revealed that these clusters presented similar features to those of FCD but were extensive and somewhat different (Hong et al., 2014). In addition, these findings suggested that extra-primary clusters may potentially be epileptogenic, as they were too subtle to discover in the visual analysis and were ignored easily, which could explain why not all patients with complete resection of the primary FCD lesion became seizure-free, especially the patients with FCD I or FCD IIa (Kwon et al., 2016).

Undoubtedly, technological advances have revolutionized the field of epilepsy in recent years. However, individual treatment according to the presurgical data was still important. For example, integration of clinical symptoms with analysis of pre-ictal EEG was conducive to establish an anatomical-electro-clinical correlation, which helped clinicians obtain the hypothesis of epileptic network. Also, substantial progress of imaging technology and computer-assistant methods was beneficial for the localization of lesions and establishment of a surgical plan. For some complex cases, in particular in surgical candidates with invisible lesions and discordant presurgical evaluation, invasive EEG technique based on a reasonable hypothesis should be preferred as it carries the advantage of allowing a three-dimensional definition of the EZ, which contributed to complete resection of the EZ and better surgical outcomes. In summary, optimization of personalized treatment was deeply connected to and dependent on the novel technology as well as the clinical information.

### Limitation

Our study had several limitations. First, as PET-CT is an expensive, radioactive medical technique, it was unethical to perform on the healthy population, so we could only include patients with HS and HC as the reference group. Herein, the neuroimaging of controls will influence the accuracy of the neural network classifier because some cases of epilepsy were dual pathology, which means that HS and FCD were combined (Chacón et al., 2008). Second, the overfitting problem discussed above may influence the final outcome. It is possible that a reduction in unnecessary components could be a solution in future research. Regularization is a way to reduce overfitting by artificially penalizing higher degree polynomials (in brief, the technique discourages learning a more complex model) (Cuingnet et al., 2013). Meanwhile, other potentially useful surface features to detect FCD may have not yet been discovered in the current literature. Third, the absence of a ground truth for each lesion label made it impossible to assess the extent to which discrepancies between manual and automated detection are errors. Additionally, we noticed the alarmingly high FP rate in the present study, which may be related to the HS controls and the low specificity of PET modality. Though the FP rate could not be completely avoided in medical testing, inclusion ideal controls and prudent consideration of PET features were helpful to alleviate it.

## Conclusion

In conclusion, automated machine learning with multimodal surface features could provide objective and intelligent detection of FCD lesion in pre-surgical evaluation and assist surgical strategy. Furthermore, the optimal parameters, appropriate surface features and efficient algorithm were worth exploring.

## Ethics Statement

All patients gave their informed consent to have their clinical and particularly neuroimaging data evaluated with regard to epilepsy research. Evaluation of clinical value was approved by the Ethics Committee of the Beijing Tiantan Hospital.

## Author Contributions

JM: acquisition of data, statistical analysis, and drafting the manuscript. WL, CC, and NZ: acquisition and interpretation of data, revising the manuscript for intellectual content. WH, CC, and YW: acquisition of data and revising the manuscript for intellectual content. XW, CL, BZ, and J-jZ: acquisition and interpretation of data. J-gZ and KZ: study design, study supervision, and final revising the manuscript for intellectual content.

## Conflict of Interest Statement

The authors declare that the research was conducted in the absence of any commercial or financial relationships that could be construed as a potential conflict of interest.

## Footnotes

1 http://www.fil.ion.ucl.ac.uk/spm/software/

2 http://surfer.nmr.mgh.harvard.edu/
